# A comprehensive method protocol for annotation and integrated functional understanding of lncRNAs

**DOI:** 10.1093/bib/bbz066

**Published:** 2019-10-03

**Authors:** Meik Kunz, Beat Wolf, Maximilian Fuchs, Jan Christoph, Ke Xiao, Thomas Thum, David Atlan, Hans-Ulrich Prokosch, Thomas Dandekar

**Affiliations:** 1 Chair of Medical Informatics, Friedrich-Alexander University of Erlangen-Nürnberg, Erlangen, Germany; 2 University of Applied Sciences and Arts of Western Switzerland, Perolles 80, 1700 Fribourg, Switzerland; 3 Functional Genomics and Systems Biology Group, Department of Bioinformatics, University of Würzburg, Germany; 4 Institute of Molecular and Translational Therapeutic Strategies (IMTTS), Hannover Medical School, Hannover, Germany; 5 REBIRTH Excellence Cluster, Hannover Medical School, Hannover, Germany; 5a National Heart and Lung Institute, Imperial College London, London, UK; 6 Phenosystems SA, 137 Rue de Tubize, 1440 Braine le Château, Belgium

**Keywords:** method protocol, lncRNAs, annotation, gene expression analysis, sequence-structure analysis, integrated functional analysis

## Abstract

Long non-coding RNAs (lncRNAs) are of fundamental biological importance; however, their functional role is often unclear or loosely defined as experimental characterization is challenging and bioinformatic methods are limited. We developed a novel integrated method protocol for the annotation and detailed functional characterization of lncRNAs within the genome. It combines annotation, normalization and gene expression with sequence-structure conservation, functional interactome and promoter analysis. Our protocol allows an analysis based on the tissue and biological context, and is powerful in functional characterization of experimental and clinical RNA-Seq datasets including existing lncRNAs. This is demonstrated on the uncharacterized lncRNA GATA6-AS1 in dilated cardiomyopathy.

## Introduction

In recent years, long non-coding RNAs (lncRNAs) have been identified as functional players that regulate important biological processes and signaling pathways associated with pathogenesis and disease progression [1–6]. Hundreds of lncRNAs were annotated, but only few are experimentally characterized due to the fact that lncRNAs have complex regulatory functions, such as binding of mRNAs, miRNAs and proteins [3, 7]. Bioinformatics approaches, such as phylogenetic and functional interaction analysis, are helpful to comprehensively understand lncRNA functions for experimental characterization [3].

Several databases, such as LNCipedia [7], NONCODE [8] and LncRBase [9], have been developed, but all these mainly provide general information about already published lncRNAs without considering the biological context. Consequently, several integrated bioinformatics tools have been designed for *de novo* analysis. Examples include starBase, which focuses on Pan-cancer analysis and interaction networks of lncRNAs based on large-scale tumor datasets [10], and the lncRNAtor tool, which helps in functional understanding of lncRNAs using data sets collected from The Cancer Genome Atlas as an example [11]. However, these tools focus on specific diseases and only provide annotated and already analyzed data from publicly available datasets. This renders them limited, especially for analysis of in-house experimental datasets as well as user-specific functional characterization of newly detected lncRNAs. Improved methods for comprehensive annotation and integrated functional understanding of lncRNAs are therefore needed.

To address this, we developed a method protocol for combined annotation and functional analysis of lncRNAs. We applied different bioinformatics approaches, such as sequence-structure conservation and regulatory network analysis, and functional context including experimentally validated genome and interactome data. The analysis protocol is innovative, time efficient and easy to use. It can therefore be used in labs as a stand-alone method for sequencing data analysis and functional characterization of lncRNAs. A detailed protocol is given in the supplementary material. Moreover, the complete workflow with all tools is implemented as a free academic trial in the Next Generation Sequencing (NGS) data analysis pipeline GensearchNGS.

## Methods

**Figure 1:**
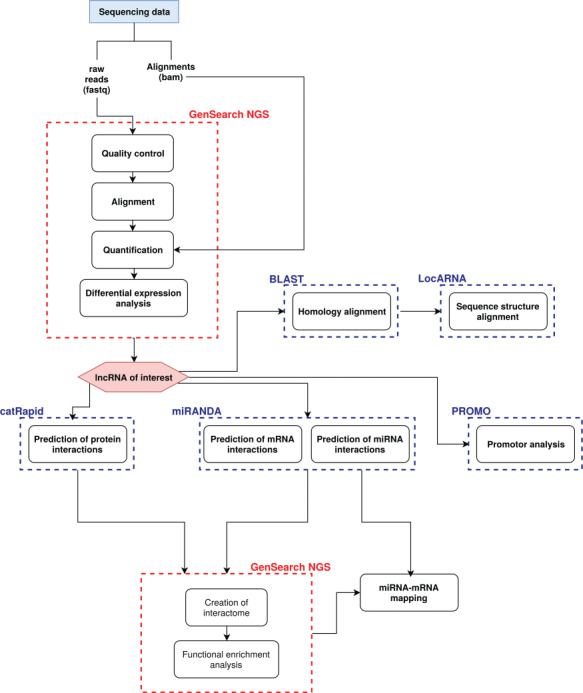
**Workflow of our method protocol.** Red dot boxes indicate the automatic modules, blue dot boxes indicate the semi-automatic modules (external reference data and programs) of the protocol flow with the analysis steps (black boxes). The input and output node indicate that this is also a stand-alone workflow.

Detailed methods are given in the supplementary material. A step-by-step protocol including Graphical User Interface (GUI) screenshot examples guides the users how to use our method, and illustrates which steps are automatically and semi-automatically implemented depending on the user interest and focus (supplementary material). Figure 1 summarizes our method protocol. It focuses on (i) annotation and gene expression analysis, (ii) sequence-structure conservation and (iii) functional analysis (see supplementary material for details).

(i) Experimental data were automatically annotated with their gene ontology (GO) terms, phenotypes and pathway associations. Read counting is based on the Gencode gene model from the Ensembl database. Expressed genes are normalized using our own implementation of the DESeq2 normalization method, whereas differential expressed genes were calculated using a t-test. The results of the gene expression analysis are shown as an interactive list, and for a more visual overview as a heatmap.
(ii) Orthologs from Ensembl and LNCipedia are used. As lncRNAs are not well conserved among species [12, 13], both databases do not provide information about lncRNA orthologous sequences. Therefore, if no orthologs are available, a BLAST analysis is implemented to manually determine the existence of orthologous sequences across species (parameters: e-value <0.01, identity ≥80%; when e-value <0.01, *P*-value and e-value are nearly identical, [14]). The resulting orthologous sequences can be uploaded to the LocARNA webserver to analyze the phylogenetic sequence-structure conservation. This gives an overview of conserved sequence and structure parts among species and thus can provide information about the interaction context.
(iii) The function analysis includes a gene enrichment analysis, promoter and regulatory network analysis.

The functional gene enrichment analysis using HPO, OMIM, Orphanet and WikiPathways helps to find overrepresented GO, pathway and phenotype terms for selected differentially expressed genes.

For network reconstruction we use experimentally validated interaction partners (mRNA, miRNA, protein) from several databases, in which a highly confident lncRNA interaction network is generated (see supplementary material). More importantly, since most lncRNAs have no experimentally validated interaction partners, potential mRNA (GATA6-AS1 sequence was split into snippets of 50 bases) and miRNA interaction partners can be predicted using miRanda [15] (parameter miRNA: scoring threshold [−sc 120]; mRNA: [−sc 160], energy [−en −72]), and protein interaction partners using catRAPID (parameter: star rating 2.5; discriminative power >75% represent high confidence predictions, [16]). We added a link to IntaRNA for further lncRNA–mRNA interaction prediction validation [17].

Furthermore, the predicted interactions can be extended using experimentally validated interaction partners from the interactome databases (String, BioGRID, CCSB and NPInter) to build up a knowledge-based interaction network. The script also allows export of the gene list, with its interactions, to Cytoscape using GML files for visualization and advanced functional network analyses. However, these various interactome annotations allow the user to interactively filter the differentially expressed genes according to criteria. Example criteria could include gene association with pathways or ontology terms, either directly or through neighboring genes on the interactome, as well as the tissue-specific expression context. This is an essential step for understanding a gene’s tissue-specific and functional role and will help users to find the best candidates for further experimentally validation.

For the promoter analysis, the script uses the lncRNA promoter sequence from −2000 to +500 bp relative to the transcription start site from Gencode and further analyzes it for transcription factor binding sites (TFBS) using a link to the Allgen PROMO tool (parameter: 0% matrix dissimilarity value to reduce false positives) [18]. Finally, the identified TFBS are combined with experimentally validated and predicted protein interaction partners. This further helps to understand the expression and complex regulatory effects of lncRNAs, for example cooperative working with an mRNA or a transcription factor (TF) in a function- and tissue-specific manner.

## Results and Discussion

To validate our protocol as an all-in-one method for lncRNA characterization, we performed analysis on a RNA-Seq dataset from GEO (GSE99321). The dataset contains seven samples from patients with dilated cardiomyopathy (DCM) and seven healthy controls. Each sample contains around 40 million reads. The expression analysis shows significant deregulation of 29 lncRNAs (Supplementary Table S1; logFC>0.75/<−0.75, *P*-value < 0.05, False Discovery Rate (FDR) adjusted *P*-value < 0.45).

In the following we focus on the experimentally uncharacterized lncRNA GATA6-AS1. The sequence-structure analysis shows a sequence-structure conservation of GATA6-AS1 among different mammals (Supplementary Figure S1; Supplementary Table S2; e-value <0.01, identity ≥80%).

lncRNAs have been implicated in a wide range of biological processes such as chromatin remodeling, recruitment of transcription factors, regulation of miRNAs, mRNA processing and translation. For this reason, the analysis of potential interaction partners of GATA6-AS1 is essential for its functional characterization. Since we are interested in the functional role of GATA6-AS1 in DCM, we focus on interactions of GATA6-AS1 with mRNAs, miRNAs and proteins, as well as their association with heart diseases. No experimentally validated interaction partners are known for GATA6-AS1. However, prediction analysis identifies 207 mRNA, 41 miRNA and 10 potential protein interaction partners, from which 7 mRNAs (SFRP1, LTBP3, SLC27A3, FAM20A, FCGBP, HS3ST2, GRB14; out of 605 mRNAs in the RNA-Seq) and 1 miRNA (miRNA-4750; out of 3 miRNAs in the RNA-Seq) show also a significant deregulation in the RNA-Seq data (Supplementary Table S3). This is a small overlap of mRNAs with the RNA-Seq data; however, it is known that interaction prediction tools are typically giving too many false positives (over-prediction). Thus, a mapping of the predicted interactions to the RNA-Seq data is always recommended and indicates strong candidate interactions for further analysis as they show an expression in the RNA-Seq.

Using the direct, experimentally validated interaction partners from NPInter and all predicted GATA6-AS1 interactors results in a knowledge-based interaction network of 2747 nodes and 66 898 edges (here not shown). Interestingly, we found an overlap with 115 RNAs from the RNA-Seq data (Supplementary Table S4). However, the functional enrichment analysis (HPO, OMIM, Orphanet, WikiPathways) of the interactome shows 55 biological pathways and phenotypes related to cardiac diseases and heart failure (Supplementary Table S5).

**Figure 2:**
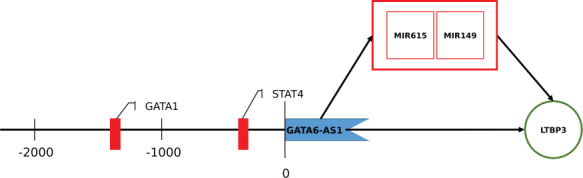
**Schematic overview of the regulatory effect of GATA6-AS1 highlighted with our method protocol.** GATA6-AS1 binds directly the mRNA LTBP3 or indirectly via sponging of miRNA-149 and miRNA-615. GATA6-AS1 can be regulated by promoter binding of GATA1 and STAT4.

To further investigate whether GATA6-AS1 indirectly regulates mRNAs via miRNA sponging, we used experimentally validated mRNA targets from NPInter of the predicted GATA6-AS1 interacting miRNAs and mapped them against the differentially expressed mRNAs from the RNA-Seq and the predicted GATA6-AS1 mRNA interactions (Supplementary Table S6). This shows 40 mRNAs, which are deregulated in the RNA-Seq and experimentally validated miRNA targets. These could be indirectly regulated by GATA6-AS1 as an effect of miRNA sponging. Moreover, 10 experimentally validated mRNA targets of the predicted miRNAs show an overlap with the predicted GATA6-AS1 mRNA interactions. Interestingly, latent transforming growth factor beta binding protein 3 (LTBP3) is also deregulated in the RNA-Seq data, which might indicate a direct regulation of GATA-AS1 via mRNA and miRNA-149 and miRNA-615 targeting (Figure 2). Further prediction validation with IntaRNA shows a potential interaction between GATA-AS1 and LTBP3, thus strengthening the probability of this interaction (Supplementary Table S6).

Finally, the promoter analysis identifies binding sites for 20 potential TFs (0% matrix dissimilarity) in the promoter of GATA6-AS1, in which heart and stress associated factors such as C/EBPbeta, AP-2alphaA, GATA-1 and STAT4 were found (Supplementary Table S7). Detailed results are given in the supplementary materials.

Together, our results suggest a functional role of GATA6-AS1 in extracellular matrix remodeling in DCM via a direct and indirect regulatory effect, e.g. targeting of LTBP3, miRNA-149 and miRNA-615, and activation through promoter binding of STAT4 and GATA1 (Figure 2). Latent-TGFβ-binding proteins such as LTBP3 are known to mediate the targeting of Transforming Growth Factor (TGF)-beta complexes into extracellular matrix structure [19, 20]. Extracellular matrix remodeling is involved in DCM progression [21], but the role of lncRNAs has not been systematically analyzed and remains still unclear [22, 23]. There is co-expression of GATA6-AS1 and LTBP3, but not regarding miRNA-149 and miRNA-615 (no independent validation dataset for co-expression analysis is available). This might be due to the fact that only three miRNAs are significantly deregulated in the RNA-Seq data. Nevertheless, our analysis strategy allows us to filter the high number of predicted interaction partners to a functional working hypothesis: GATA6-AS1 interactions involve the deregulated and predicted LTBP3 as well as miRNA-149 and miRNA-615 as predicted GATA6-AS1 miRNAs and known experimentally validated LTBP3 targets. These demonstration results need to be analyzed by validation experiments to examine the role of GAT6-AS1 in DCM. The sequence-structure conservation of GATA6-AS1 in mammals allows development of experimental models.

Interestingly, a recent paper also supports a role of GATA6-AS1 in cardiac development. This was demonstrated by a computational method that detects triplex-forming lncRNAs and DNA targets [24]. This independently supports the ability of our tool to functional characterize lncRNAs.

Notably, our scripts can also be used for analysis of other RNAs, for instance for mRNAs, in particular when combined with suitable refined analysis steps (in particular separate analysis of 5´UTR, CDS and 3´UTR for motifs). We tested this on several miRNAs and mRNAs (examples include [25–27]). As our approach is generic and modular implemented, the protocol is even suitable for genomic analysis to identify variants relevant for disease development, e.g. heart and tumor. Moreover, the method pipeline could be integrated in the IT infrastructure of data integration centers of university hospitals, or molecular tumor boards to improve the university medicine [28].

### Study limitations

Our aim was to develop a general method protocol for lncRNA analysis and functional characterization that is not restricted to specific disease types. We focus on quite common lncRNA interactions with RNAs (miRNAs, mRNAs) and proteins, but we want to point out that more mechanistic interactions exist to describe the complex lncRNA effects [29]. The modularity of our protocol allows to extend it for further analysis depending on the interests of the users, for instance regarding R-loop binding (e.g. R-loopDB [30]) and RNA:DNA triplex formation (several RNA–RNA and RNA–DNA interaction prediction tools can be found in [31]). This makes the protocol useful to a broad community.

RNA sequencing analysis always raises questions regarding sample size and power estimation. Considering different experimental settings that influence the statistical power, we are using DESeq2 for differential expression analysis, which is known to give high performance power, also when the sample size is ≤5 [32]. We applied multiple-testing correction using the FDR method. We used as cut-offs *P*-value < 0.05 and FDR adjusted *P*-value < 0.45. Since high-throughput sequencing methods such as RNA-Seq bear a high error rate, in particular due to sample preparation, small sample sizes and heterogeneous data from adult and young patient cohorts, detection of differential expressed genes remains difficult. For instance, many genes detected with low FDR adjusted *P*-value cannot be validated in experiments [33]. In this context, to avoid underestimation of FDRs we used a *P*-value < 0.05 as significant cut-off criteria to select top candidates for further comprehensive functional characterization in independent low-throughput approaches.

Moreover, it is also challenging to estimate power and satisfactory sample size, especially for lncRNAs that show lower expression and power relative to mRNAs from the same experiment (extensively reviewed in [32]). As the power and sample size from experiments directly influence the conclusions from our analysis protocol, users are alerted that an analysis pipeline is no substitute for good quality data and functional validation experiments. We recommend that users should carefully plan their experiments e.g. using approaches such as paired-sample RNA-Seq and multifactor experimental design, which significantly enhances the statistical power (e.g. use the RNASeqPowerCalculator [32] to estimate the sample size).

Conservation analysis is widely used for functional characterization of newly discovered lncRNAs. Unlike protein-coding genes and miRNAs, lncRNAs are highly variable and less conserved among species, thus hampering translational approaches [12, 29, 34–36]. For instance, lncRNA functional conservation can be obtained across large evolutionary distances even with no sequence and structure similarity [12]. This multifaceted levels of conservation was demonstrated for the well-known lncRNAs Malat1 and Hotair [13]. Therefore, lncRNA characterization should focus on sequence, structure and function analysis [12, 13]. As most lncRNAs are functional uncharacterized, we integrated approaches such as interaction prediction, which have per se limitations regarding reliability and specificity as they typically tend to over-predictions. Moreover, prediction tools require significant amount of computational power especially for lncRNA interaction analysis with whole transcriptomes. For this, we used miRanda after advantageously splitting lncRNAs into snippets of 50 nt to reduce the computational burden of lncRNA–mRNA interaction and secondary structure calculation. However, we also tried tools such as IntaRNA, but the computational burden was too high for the whole transcriptome. Similarly, studies from Antonov *et al*. [31] compared different lncRNA-mRNA prediction tools, but limited the analysis on 17 interactions between transcripts shorter than 5000 nt due to the computation burden and limited computation power. We know that our strategy raises potential problems as the secondary structure of lncRNAs is instrumental for the function, whereas prediction tools such miRanda are based on seed base-pairing and take secondary structure, hence only quite locally into account. However, several functional lncRNAs lack sequence and structural conservation [12, 31]. In this context, our aim was to allow prediction of lncRNA interactions with whole transcriptomes, but this strategy needs further validation steps as included in our pipeline. For instance, mapping of the predicted interactions to the RNA-Seq data indicates strong candidate interactions for further analysis as they show an expression in the RNA-Seq data. Moreover, further validation of the mRNA interaction candidates with general lncRNA–mRNA prediction tools such as IntaRNA will strengthen the interaction prediction accuracy (several prediction tools can be found in [31]). As an alternative to the whole transcriptome approach with miRanda, users can calculate lncRNA–mRNA interactions with a defined set of mRNA transcripts (e.g. well-known mRNAs for cardiac disease) using IntaRNA.

Taken together, we combine several analyses and filtering steps (sequence, structure, functional interaction context) to comprehensively investigate the regulatory role of the identified lncRNAs. In our example dataset we used stringent thresholds e.g. for e-value [14] and prediction power [16] as recommended for best quality prediction accuracy from these programs. Nevertheless, analysis can be performed also using different cut-offs (high/low stringent). This different analysis steps and filter cut-offs strengthen the robustness and reduce false positive, thus allowing to select most reliable candidates for further experimental testing. We applied this analysis strategy as useful software and support in testing RNA functionalities for the hypertrophy associated lncRNA Chast [6] and human hypoxia-sensitive lncRNAs LINC00323–003 and MIR503HG [29].

## Conclusion

In conclusion, we present a fast and efficient all-in-one method for annotation and functional analysis of lncRNAs. This is the first time that a method protocol follows such a comprehensive analysis strategy, combining sequence-structure conservation analysis and promoter analysis with the functional interaction context. Nevertheless, lncRNA analysis is challenging and the reader is alerted that a good analysis pipeline is no substitute for more good quality data and more functional validation experiments. In-depth studies are always required to fully understand and describe complex lncRNA functions and effects. However, we believe that our method is a powerful help in detailed understanding of the complex regulatory effects of lncRNAs within the genome. This in turn could open new windows for effective clinical strategies for better management of diseases. Our protocol works for uncharacterized as well as already experimentally characterized lncRNAs. Thus, our functional analysis strategy will help to better elucidate the complex regulatory effect of lncRNAs and is a powerful method protocol for daily scientific work.

## Data availability

The method protocol and source code are available in the supplementary material. Moreover, the protocol is implemented as an automatic and user-interactive graphical workflow in the NGS data analysis pipeline GensearchNGS available as a free academic trial under the link http://www.phenosystems.com.

**Key Points**

- lncRNAs play an important biological role; however, the functional role is often unclear as experimental characterization is challenging and bioinformatic methods focus on specific diseases and mainly provide general information.
- We developed a powerful all-in-one method protocol for detailed understanding of the complex regulatory effects of lncRNAs combining sequence-structure conservation analysis and promoter analysis with the functional interaction context.
- The protocol allows analysis of experimental datasets including already experimental characterized as well as uncharacterized lncRNAs.
- It considers the tissue and biological context, and works for several disease types.

## Supplementary Material

Supplementary_Figure_S1A_bbz066

Supplementary_Figure_S1B_bbz066

Supplementary_Table_S1_2nd_revision_bbz066

Supplementary_Table_S2_bbz066

Supplementary_Table_S3_bbz066

Supplementary_Table_S4_bbz066

Supplementary_Table_S5_bbz066

Supplementary_Table_S6_2nd_revision_bbz066

Supplementary_Table_S7_bbz066

## Notes

**Meik Kunz** is group leader at the Chair of Medical Informatics, Friedrich-Alexander University of Erlangen-Nürnberg, Erlangen, Germany.

**Beat Wolf** is postdoc at the University of Applied Sciences and Arts of Western Switzerland, Fribourg, Switzerland.

**Maximilian Fuchs** is PhD student at the Department of Bioinformatics, University of Würzburg, Germany.

**Jan Christoph** is postdoc at the Chair of Medical Informatics, Friedrich-Alexander University of Erlangen-Nürnberg, Erlangen, Germany.

**Ke Xiao** is group leader at the Institute of Molecular and Translational Therapeutic Strategies, Hannover Medical School, Hannover, Germany.

**Thomas Thum** is chair and full professor at the Institute of Molecular and Translational Therapeutic Strategies and member of the REBIRTH Excellence Cluster (Hannover Medical School, Hannover, Germany) as well as visiting professor at the National Heart and Lung Institute, Imperial College London, London, UK.

**David Atlan** is CEO of the NGS data analysis company Phenosystems SA, Braine le Château, Belgium.

**Hans-Ulrich Prokosch** is chair and full professor at the Chair of Medical Informatics, Friedrich-Alexander University of Erlangen-Nürnberg, Erlangen, Germany.

**Thomas Dandekar** is chair and full professor at the Department of Bioinformatics, University of Würzburg, Germany.
