# Supplementary figures and images for: A comprehensive method protocol for annotation and integrated functional understanding of lncRNAs
Source: Brief Bioinform. 2019 Oct 3;21(4):1391–6. doi: 10.1093/bib/bbz066 (PMC7373182; doi:10.1093/bib/bbz066)

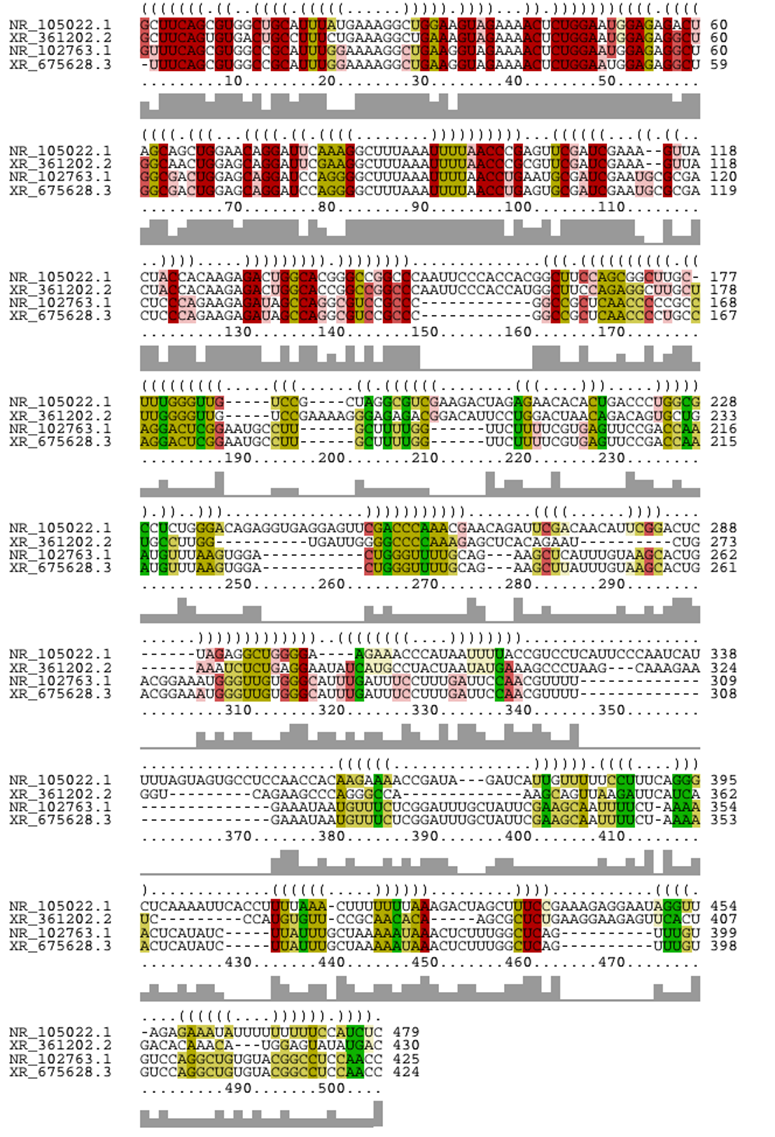

Supplement: Supplementary_Figure_S1A_bbz066 [file supplementary_figure_s1a_bbz066.png]

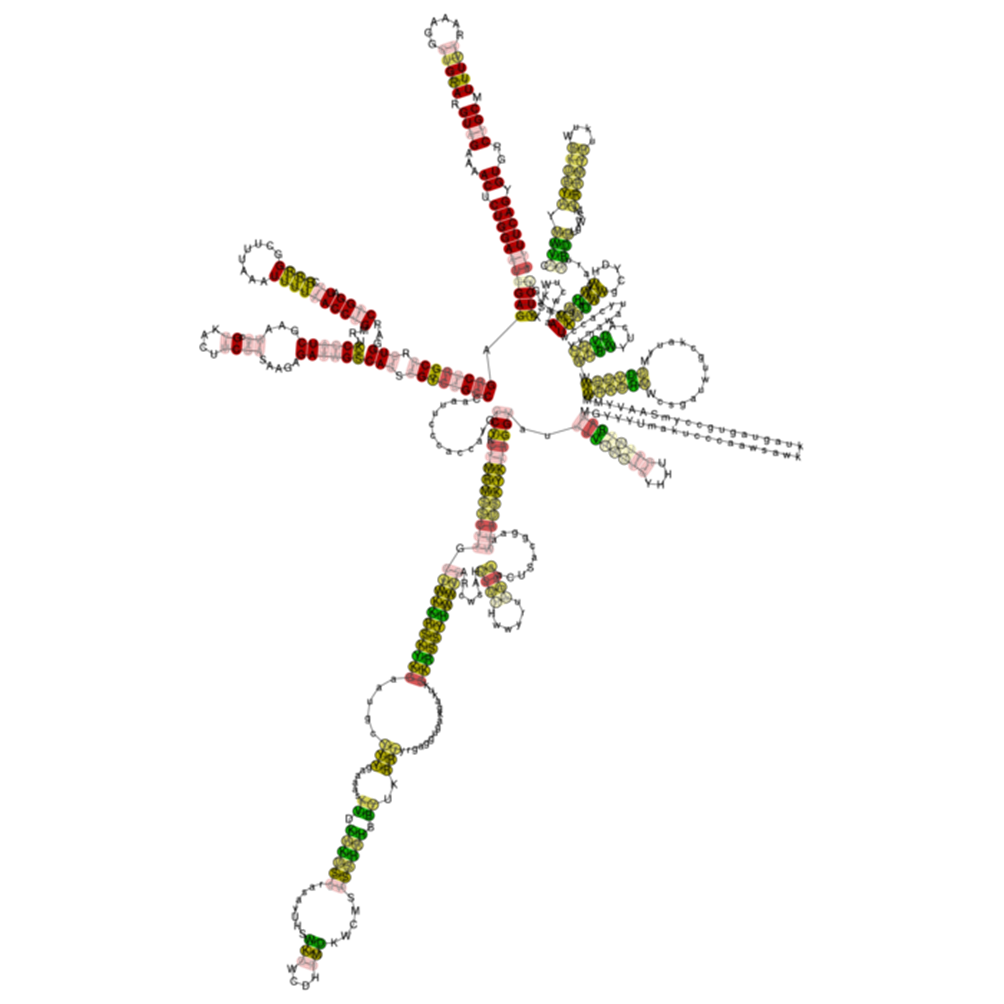

Supplement: Supplementary_Figure_S1B_bbz066 [file supplementary_figure_s1b_bbz066.docx]
