# Supplementary material for: A comprehensive method protocol for annotation and integrated functional understanding of lncRNAs
Source: Brief Bioinform. 2019 Oct 3;21(4):1391–6. doi: 10.1093/bib/bbz066 (PMC7373182; doi:10.1093/bib/bbz066)
Supplement: Supplementary_Table_S2_bbz066 [file supplementary_table_s2_bbz066.docx]

| **Species Name** | **NCBI Accession** | **Identity (%)** | **e-value** |
| --- | --- | --- | --- |
| Homo sapiens | NR_102763.1 | 100 | 0.0 |
| Pan troglodytes | XR_675628.3 | 98.5 | 0.0 |
| Rattus norvegicus | XR_361202.2 | 81.6 | 4.49e-24 |
| Mus musculus | NR_105022.1 | 81.6 | 4.49e-24 |
