# Supplementary material for: A comprehensive method protocol for annotation and integrated functional understanding of lncRNAs
Source: Brief Bioinform. 2019 Oct 3;21(4):1391–6. doi: 10.1093/bib/bbz066 (PMC7373182; doi:10.1093/bib/bbz066)
Supplement: Supplementary_Table_S3_bbz066 [file supplementary_table_s3_bbz066.docx]

| **Name** | **Type** |
| --- | --- |
| MIR6732 | microRNA |
| MIR4632 | microRNA |
| MIR4787 | microRNA |
| MIR939 | microRNA |
| MIR6749 | microRNA |
| MIR4539 | microRNA |
| MIR1180 | microRNA |
| MIR6787 | microRNA |
| MIR6776 | microRNA |
| MIR181D | microRNA |
| MIR6793 | microRNA |
| MIR1227 | microRNA |
| MIR4750 | microRNA |
| MIR6751 | microRNA |
| MIR1225 | microRNA |
| MIR762 | microRNA |
| MIR6080 | microRNA |
| MIR6821 | microRNA |
| MIR6802 | microRNA |
| MIR6752 | microRNA |
| MIR6835 | microRNA |
| MIR6850 | microRNA |
| MIR6756 | microRNA |
| MIR6716 | microRNA |
| MIR6765 | microRNA |
| MIR5196 | microRNA |
| MIR149 | microRNA |
| MIR6088 | microRNA |
| MIR615 | microRNA |
| MIR4689 | microRNA |
| MIR6089 | microRNA |
| MIR3960 | microRNA |
| MIR2392 | microRNA |
| MIR6794 | microRNA |
| MIR1268B | microRNA |
| MIR4433A | microRNA |
| MIR4443 | microRNA |
| MIR302C | microRNA |
| MIR4433B | microRNA |
| MIR4516 | microRNA |
| MIR4466 | microRNA |
| ADAM15 | Protein coding (mRNA) |
| ADAMTS7 | Protein coding (mRNA) |
| ADGRA2 | Protein coding (mRNA) |
| ADRA1B | Protein coding (mRNA) |
| AGAP1 | Protein coding (mRNA) |
| AGAP3 | Protein coding (mRNA) |
| AJAP1 | Protein coding (mRNA) |
| AMH | Protein coding (mRNA) |
| ANKRD65 | Protein coding (mRNA) |
| ANKRD9 | Protein coding (mRNA) |
| APC2 | Protein coding (mRNA) |
| APPL2 | Protein coding (mRNA) |
| ARHGAP23 | Protein coding (mRNA) |
| ARL4C | Protein coding (mRNA) |
| ATP11C | Protein coding (mRNA) |
| ATP2A3 | Protein coding (mRNA) |
| ATXN2 | Protein coding (mRNA) |
| BAHCC1 | Protein coding (mRNA) |
| BCL3 | Protein coding (mRNA) |
| BHLHE41 | Protein coding (mRNA) |
| BRF1 | Protein coding (mRNA) |
| BRSK2 | Protein coding (mRNA) |
| BX255925.3 | Protein coding (mRNA) |
| C11orf96 | Protein coding (mRNA) |
| C2CD3 | Protein coding (mRNA) |
| CASK | Protein coding (mRNA) |
| CAST | Protein coding (mRNA) |
| CCDC92 | Protein coding (mRNA) |
| CCNE1 | Protein coding (mRNA) |
| CDH22 | Protein coding (mRNA) |
| CDK5R2 | Protein coding (mRNA) |
| CDT1 | Protein coding (mRNA) |
| CEBPD | Protein coding (mRNA) |
| CENPB | Protein coding (mRNA) |
| CENPVL1 | Protein coding (mRNA) |
| CENPVL2 | Protein coding (mRNA) |
| CGB7 | Protein coding (mRNA) |
| CHGA | Protein coding (mRNA) |
| CHST1 | Protein coding (mRNA) |
| CHST2 | Protein coding (mRNA) |
| CLSTN1 | Protein coding (mRNA) |
| CMTM8 | Protein coding (mRNA) |
| CPLX1 | Protein coding (mRNA) |
| DDHD2 | Protein coding (mRNA) |
| DVL1 | Protein coding (mRNA) |
| EHBP1L1 | Protein coding (mRNA) |
| EPB41L4B | Protein coding (mRNA) |
| EPOP | Protein coding (mRNA) |
| EPPK1 | Protein coding (mRNA) |
| ESPN | Protein coding (mRNA) |
| EVX2 | Protein coding (mRNA) |
| F8A1 | Protein coding (mRNA) |
| F8A2 | Protein coding (mRNA) |
| F8A3 | Protein coding (mRNA) |
| FAM102B | Protein coding (mRNA) |
| FAM120C | Protein coding (mRNA) |
| FAM171A2 | Protein coding (mRNA) |
| FAM20A | Protein coding (mRNA) |
| FASN | Protein coding (mRNA) |
| FBXO41 | Protein coding (mRNA) |
| FCGBP | Protein coding (mRNA) |
| FERMT1 | Protein coding (mRNA) |
| FIGNL2 | Protein coding (mRNA) |
| FIZ1 | Protein coding (mRNA) |
| FMNL1 | Protein coding (mRNA) |
| FOXD2 | Protein coding (mRNA) |
| FOXE3 | Protein coding (mRNA) |
| FOXL2 | Protein coding (mRNA) |
| GABBR2 | Protein coding (mRNA) |
| GABRD | Protein coding (mRNA) |
| GMPS | Protein coding (mRNA) |
| GRB14 | Protein coding (mRNA) |
| GRIN2D | Protein coding (mRNA) |
| GRSF1 | Protein coding (mRNA) |
| GXYLT2 | Protein coding (mRNA) |
| HCN2 | Protein coding (mRNA) |
| HCN4 | Protein coding (mRNA) |
| HDAC4 | Protein coding (mRNA) |
| HELZ2 | Protein coding (mRNA) |
| HES2 | Protein coding (mRNA) |
| HES4 | Protein coding (mRNA) |
| HOXB4 | Protein coding (mRNA) |
| HS3ST2 | Protein coding (mRNA) |
| HS3ST4 | Protein coding (mRNA) |
| IFT27 | Protein coding (mRNA) |
| IGFN1 | Protein coding (mRNA) |
| ILDR2 | Protein coding (mRNA) |
| IRF2BPL | Protein coding (mRNA) |
| IRS2 | Protein coding (mRNA) |
| IRX2 | Protein coding (mRNA) |
| ITGB2 | Protein coding (mRNA) |
| JPH4 | Protein coding (mRNA) |
| KCNA7 | Protein coding (mRNA) |
| KCNE5 | Protein coding (mRNA) |
| KCNH3 | Protein coding (mRNA) |
| KCNMB4 | Protein coding (mRNA) |
| KCNQ1 | Protein coding (mRNA) |
| KCNQ2 | Protein coding (mRNA) |
| KLF16 | Protein coding (mRNA) |
| KLHL21 | Protein coding (mRNA) |
| KLHL35 | Protein coding (mRNA) |
| LEMD2 | Protein coding (mRNA) |
| LETM1 | Protein coding (mRNA) |
| LINGO3 | Protein coding (mRNA) |
| LRFN3 | Protein coding (mRNA) |
| LRFN4 | Protein coding (mRNA) |
| LRRN4CL | Protein coding (mRNA) |
| LTBP3 | Protein coding (mRNA) |
| MAGI2 | Protein coding (mRNA) |
| MAP3K1 | Protein coding (mRNA) |
| MAP3K6 | Protein coding (mRNA) |
| MDGA1 | Protein coding (mRNA) |
| MFHAS1 | Protein coding (mRNA) |
| MFSD14B | Protein coding (mRNA) |
| MGAT1 | Protein coding (mRNA) |
| MGAT4B | Protein coding (mRNA) |
| MGAT5B | Protein coding (mRNA) |
| MIB2 | Protein coding (mRNA) |
| MIDN | Protein coding (mRNA) |
| MSX1 | Protein coding (mRNA) |
| MXI1 | Protein coding (mRNA) |
| MYO1D | Protein coding (mRNA) |
| NAT8L | Protein coding (mRNA) |
| NDFIP1 | Protein coding (mRNA) |
| NIT2 | Protein coding (mRNA) |
| NKX1-1 | Protein coding (mRNA) |
| NKX1-2 | Protein coding (mRNA) |
| NKX2-8 | Protein coding (mRNA) |
| NKX3-2 | Protein coding (mRNA) |
| NKX6-1 | Protein coding (mRNA) |
| NLGN2 | Protein coding (mRNA) |
| NOTUM | Protein coding (mRNA) |
| NOVA2 | Protein coding (mRNA) |
| NPAS2 | Protein coding (mRNA) |
| NPTXR | Protein coding (mRNA) |
| NR2F6 | Protein coding (mRNA) |
| OSBPL1A | Protein coding (mRNA) |
| P2RY6 | Protein coding (mRNA) |
| PACS2 | Protein coding (mRNA) |
| PAPPA | Protein coding (mRNA) |
| PCYT2 | Protein coding (mRNA) |
| PDE10A | Protein coding (mRNA) |
| PGR | Protein coding (mRNA) |
| PHF2 | Protein coding (mRNA) |
| PHOSPHO1 | Protein coding (mRNA) |
| PIGS | Protein coding (mRNA) |
| PKD1 | Protein coding (mRNA) |
| PLCG1 | Protein coding (mRNA) |
| PLPPR3 | Protein coding (mRNA) |
| PLPPR5 | Protein coding (mRNA) |
| PLXND1 | Protein coding (mRNA) |
| PPM1L | Protein coding (mRNA) |
| RAPGEF2 | Protein coding (mRNA) |
| RASSF7 | Protein coding (mRNA) |
| RBMXL2 | Protein coding (mRNA) |
| RNPEPL1 | Protein coding (mRNA) |
| SAMD1 | Protein coding (mRNA) |
| SFRP1 | Protein coding (mRNA) |
| SH2B2 | Protein coding (mRNA) |
| SHANK3 | Protein coding (mRNA) |
| SHB | Protein coding (mRNA) |
| SHH | Protein coding (mRNA) |
| SHISA7 | Protein coding (mRNA) |
| SKOR2 | Protein coding (mRNA) |
| SLAIN2 | Protein coding (mRNA) |
| SLC16A8 | Protein coding (mRNA) |
| SLC22A23 | Protein coding (mRNA) |
| SLC22A5 | Protein coding (mRNA) |
| SLC25A6 | Protein coding (mRNA) |
| SLC27A3 | Protein coding (mRNA) |
| SLC2A4RG | Protein coding (mRNA) |
| SOCS3 | Protein coding (mRNA) |
| SOX21 | Protein coding (mRNA) |
| SOX4 | Protein coding (mRNA) |
| SP8 | Protein coding (mRNA) |
| TALDO1 | Protein coding (mRNA) |
| TBC1D12 | Protein coding (mRNA) |
| TBC1D4 | Protein coding (mRNA) |
| TBR1 | Protein coding (mRNA) |
| TCF3 | Protein coding (mRNA) |
| TENT4A | Protein coding (mRNA) |
| TF | Protein coding (mRNA) |
| TFAP2E | Protein coding (mRNA) |
| TMEM255B | Protein coding (mRNA) |
| TNRC18 | Protein coding (mRNA) |
| TNS1 | Protein coding (mRNA) |
| TPGS1 | Protein coding (mRNA) |
| TRIM8 | Protein coding (mRNA) |
| TSC2 | Protein coding (mRNA) |
| TSHZ1 | Protein coding (mRNA) |
| TYMS | Protein coding (mRNA) |
| UBALD2 | Protein coding (mRNA) |
| UBE2F | Protein coding (mRNA) |
| UTF1 | Protein coding (mRNA) |
| VGLL4 | Protein coding (mRNA) |
| WDR81 | Protein coding (mRNA) |
| XYLT1 | Protein coding (mRNA) |
| YTHDF1 | Protein coding (mRNA) |
| ZCCHC14 | Protein coding (mRNA) |
| ZFPM1 | Protein coding (mRNA) |
| ZMIZ1 | Protein coding (mRNA) |
| ZNF316 | Protein coding (mRNA) |
| ZNF503 | Protein coding (mRNA) |
| ZNF697 | Protein coding (mRNA) |
| ZNF865 | Protein coding (mRNA) |
| ZNRF2 | Protein coding (mRNA) |
| ZZEF1 | Protein coding (mRNA) |
| SFPQ | Protein |
| PTBP1 | Protein |
| TIA1 | Protein |
| ELAVL1 | Protein |
| ELAVL3 | Protein |
| HNRNPA1 | Protein |
| ELAVL2 | Protein |
| KHDRBS3 | Protein |
| ESRP2 | Protein |
| U2AF2 | Protein |
