# Supplementary material for: A comprehensive method protocol for annotation and integrated functional understanding of lncRNAs
Source: Brief Bioinform. 2019 Oct 3;21(4):1391–6. doi: 10.1093/bib/bbz066 (PMC7373182; doi:10.1093/bib/bbz066)
Supplement: Supplementary_Table_S4_bbz066 [file supplementary_table_s4_bbz066.docx]

| **Chr.** | **Name** | **logFC** | **Ref seq.** | **Tested seq.** | **Total seq.** | **p-value** | **Coding type** |
| --- | --- | --- | --- | --- | --- | --- | --- |
| 13 | ALG5 | -0,896 | 26,006 | 13,97 | 925 | 0,028 | Protein coding |
| 4 | ANAPC10 | -0,817 | 26,102 | 14,817 | 718 | 0,044 | Protein coding |
| 5 | ANKHD1-EIF4EBP3 | 2,509 | 1,644 | 9,358 | 82 | 0,039 | Protein coding |
| 2 | ANKRD23 | 1,799 | 12,45 | 43,329 | 1368 | 0,01 | Protein coding |
| 18 | ANKRD29 | 1,124 | 12,46 | 27,159 | 940 | 0,012 | Protein coding |
| 3 | ARIH2 | 0,87 | 401,836 | 734,367 | 25962 | 0,002 | Protein coding |
| 1 | ATP1A1 | -1,094 | 1.893,36 | 887,116 | 61067 | 0,026 | Protein coding |
| 10 | ATRNL1 | 4,046 | 0,65 | 10,737 | 246 | 0,003 | Protein coding |
| 12 | BCAT1 | -1,208 | 43,149 | 18,68 | 915 | 0,008 | Protein coding |
| 11 | CARS | -0,864 | 73,321 | 40,275 | 2297 | 0,02 | Protein coding |
| 13 | CCDC122 | 1,457 | 8,028 | 22,044 | 449 | 0,022 | Protein coding |
| 5 | CCNG1 | -1,023 | 275,383 | 135,497 | 9325 | 0,006 | Protein coding |
| 4 | CCNG2 | 1,035 | 24,479 | 50,174 | 1749 | 0,028 | Protein coding |
| 4 | CCSER1 | 1,768 | 4,086 | 13,911 | 300 | 0,007 | Protein coding |
| 14 | CDKN3 | -2,116 | 5,379 | 1,241 | 185 | 0,018 | Protein coding |
| 5 | CENPH | 1,181 | 3,037 | 6,884 | 243 | 0,041 | Protein coding |
| 2 | CHN1 | 0,773 | 28,6 | 48,87 | 1678 | 0,038 | Protein coding |
| 1 | CLIC4 | -1,067 | 1.835,22 | 876,217 | 61290 | 0 | Protein coding |
| X | COL4A5 | 0,82 | 32,386 | 57,155 | 1728 | 0,004 | Protein coding |
| 16 | COTL1 | -1,404 | 82,326 | 31,102 | 2387 | 0,006 | Protein coding |
| 4 | CPLX1 | 0,884 | 8,67 | 16,001 | 487 | 0,045 | Protein coding |
| 17 | DBF4B | 1,372 | 3,886 | 10,062 | 318 | 0,005 | Protein coding |
| 4 | DHX15 | -0,828 | 226,937 | 127,814 | 7659 | 0,026 | Protein coding |
| 19 | DMKN | 2,031 | 8,865 | 36,227 | 737 | 0,014 | Protein coding |
| 9 | DOLPP1 | -1,03 | 16,598 | 8,129 | 676 | 0,039 | Protein coding |
| 19 | DPY19L3 | -0,923 | 45,391 | 23,937 | 1514 | 0,036 | Protein coding |
| 1 | DTL | -1,665 | 3,024 | 0,954 | 107 | 0,043 | Protein coding |
| 1 | ECHDC2 | 0,866 | 101,738 | 185,458 | 6929 | 0,006 | Protein coding |
| 14 | EFCAB11 | 1,966 | 2,167 | 8,465 | 226 | 0,007 | Protein coding |
| 3 | EIF4G1 | -0,946 | 1.138,54 | 591,14 | 39330 | 0,008 | Protein coding |
| 19 | EMP3 | -0,95 | 87,294 | 45,182 | 3103 | 0,014 | Protein coding |
| 4 | FAM160A1 | -1,549 | 29,29 | 10,009 | 775 | 0,024 | Protein coding |
| 17 | FAM20A | 1,116 | 23,739 | 51,436 | 1155 | 0,031 | Protein coding |
| 8 | FAM91A1 | -0,98 | 50,748 | 25,726 | 1635 | 0,022 | Protein coding |
| 19 | FCGBP | -3,744 | 28,459 | 2,124 | 974 | 0,031 | Protein coding |
| 12 | FKBP11 | -1,088 | 15,485 | 7,283 | 620 | 0,041 | Protein coding |
| 17 | FOXK2 | -0,884 | 173,047 | 93,752 | 6089 | 0,015 | Protein coding |
| 3 | GNL3 | -1,179 | 238,359 | 105,253 | 4681 | 0,042 | Protein coding |
| 2 | GRB14 | -1,133 | 31,534 | 14,377 | 1035 | 0,039 | Protein coding |
| 7 | GTPBP10 | 0,854 | 33,25 | 60,091 | 1608 | 0,039 | Protein coding |
| 4 | H2AFZ | -1,141 | 131,097 | 59,455 | 5373 | 0,032 | Protein coding |
| 14 | HIF1A | -1,371 | 444,07 | 171,66 | 12035 | 0,031 | Protein coding |
| 2 | HJURP | -4,987 | 6,066 | 0,191 | 100 | 0,015 | Protein coding |
| 16 | HS3ST2 | -2,1 | 1,169 | 0,273 | 50 | 0,038 | Protein coding |
| 3 | IGF2BP2 | -1,015 | 50,152 | 24,82 | 1618 | 0,019 | Protein coding |
| 7 | IKZF1 | -1,369 | 12,601 | 4,879 | 472 | 0,042 | Protein coding |
| 7 | IMMP2L | 0,847 | 14,451 | 25,999 | 785 | 0,028 | Protein coding |
| 3 | IQCG | -1,825 | 55,992 | 15,809 | 934 | 0,044 | Protein coding |
| 18 | KIAA1328 | 1,522 | 8,807 | 25,289 | 603 | 0,007 | Protein coding |
| 6 | KIF13A | 0,769 | 262,983 | 448,079 | 16083 | 0,006 | Protein coding |
| 2 | KLHL23 | -0,92 | 72,455 | 38,29 | 2096 | 0,031 | Protein coding |
| 2 | LPIN1 | -0,81 | 194,357 | 110,832 | 7031 | 0,029 | Protein coding |
| 11 | LTBP3 | 0,955 | 225,192 | 436,504 | 18522 | 0,028 | Protein coding |
| 1 | MARK1 | -0,753 | 68,036 | 40,37 | 2393 | 0,028 | Protein coding |
| 14 | MARK3 | -1,161 | 364,488 | 162,978 | 11619 | 0,002 | Protein coding |
| 2 | MEIS1 | -0,912 | 153,782 | 81,743 | 4749 | 0,008 | Protein coding |
| 2 | METTL8 | -0,892 | 21,471 | 11,569 | 784 | 0,047 | Protein coding |
| 5 | MFAP3 | -1,453 | 50,46 | 18,427 | 1422 | 0,018 | Protein coding |
| 12 | MGST1 | -2,53 | 46,546 | 8,059 | 1687 | 0,017 | Protein coding |
| 17 | MKS1 | 0,915 | 11,158 | 21,032 | 829 | 0,038 | Protein coding |
| 3 | MRPL3 | -0,83 | 128,597 | 72,321 | 4586 | 0,013 | Protein coding |
| 4 | MTHFD2L | 1,428 | 10,347 | 27,834 | 723 | 0,021 | Protein coding |
| 15 | MYO9A | -0,916 | 123,931 | 65,676 | 3651 | 0,021 | Protein coding |
| 5 | NDUFAF2 | -0,871 | 42,158 | 23,048 | 1316 | 0,041 | Protein coding |
| 15 | NEDD4 | -0,777 | 44,123 | 25,744 | 1422 | 0,027 | Protein coding |
| 11 | PAAF1 | 1,528 | 24,41 | 70,411 | 1705 | 0,003 | Protein coding |
| 12 | PAN2 | 0,798 | 135,405 | 235,378 | 5480 | 0,029 | Protein coding |
| 12 | PARPBP | -2,332 | 3,612 | 0,718 | 77 | 0,025 | Protein coding |
| 21 | PCNT | -0,939 | 330,679 | 172,465 | 9370 | 0,023 | Protein coding |
| 4 | PI4K2B | -1,035 | 16,798 | 8,2 | 598 | 0,03 | Protein coding |
| 6 | PLAGL1 | 0,893 | 67,176 | 124,716 | 4937 | 0,042 | Protein coding |
| 7 | PNPLA8 | -1,058 | 189,586 | 91,04 | 6285 | 0,036 | Protein coding |
| 10 | PPA1 | -0,899 | 124,064 | 66,511 | 4735 | 0,024 | Protein coding |
| 20 | PPDPF | 1,234 | 205,837 | 484,143 | 19293 | 0,024 | Protein coding |
| 2 | PSMD14 | -0,821 | 131,179 | 74,24 | 4197 | 0 | Protein coding |
| 1 | PTP4A2 | -1,195 | 541,952 | 236,775 | 18124 | 0,005 | Protein coding |
| 1 | RABGAP1L | 0,916 | 178,366 | 336,57 | 11208 | 0,002 | Protein coding |
| 9 | RCL1 | -0,907 | 31,86 | 16,989 | 914 | 0,028 | Protein coding |
| 1 | RGS2 | -0,884 | 70,774 | 38,338 | 2343 | 0,047 | Protein coding |
| 6 | RNASET2 | 1,274 | 12,305 | 29,755 | 835 | 0,01 | Protein coding |
| 1 | RPAP2 | -0,952 | 51,515 | 26,628 | 1480 | 0,024 | Protein coding |
| 4 | RPL9 | -1,557 | 49,423 | 16,791 | 1487 | 0,027 | Protein coding |
| 16 | RSPRY1 | -0,865 | 61,982 | 34,035 | 2201 | 0,005 | Protein coding |
| 8 | SDCBP | -0,907 | 415,827 | 221,775 | 15312 | 0,042 | Protein coding |
| 8 | SFRP1 | 2,102 | 73,213 | 314,384 | 10403 | 0,003 | Protein coding |
| 9 | SH3GL2 | 1,788 | 6,535 | 22,572 | 754 | 0,006 | Protein coding |
| 5 | SLC1A3 | -0,885 | 335,11 | 181,5 | 10379 | 0,025 | Protein coding |
| 1 | SLC27A3 | 1,022 | 21,9 | 44,48 | 1738 | 0,03 | Protein coding |
| 11 | SLC36A4 | -1,743 | 26,761 | 7,993 | 901 | 0,004 | Protein coding |
| 4 | SMIM14 | 0,927 | 42,91 | 81,594 | 2154 | 0,004 | Protein coding |
| 4 | SORBS2 | 0,866 | 1.952,53 | 3.559,33 | 128504 | 0,017 | Protein coding |
| 3 | SRGAP3 | 1,189 | 10,996 | 25,074 | 807 | 0,006 | Protein coding |
| 10 | SRGN | -1,171 | 194,893 | 86,57 | 6984 | 0,012 | Protein coding |
| 17 | STAT3 | -1,847 | 1.028,98 | 286,049 | 29990 | 0,022 | Protein coding |
| X | TAZ | 0,83 | 95,834 | 170,327 | 4777 | 0,013 | Protein coding |
| 3 | TFRC | -1,042 | 391,167 | 189,942 | 9942 | 0,022 | Protein coding |
| 17 | TMC6 | 0,889 | 103,89 | 192,393 | 6315 | 0,009 | Protein coding |
| 2 | TPRKB | -0,895 | 65,081 | 34,997 | 1573 | 0,035 | Protein coding |
| 6 | TRMT11 | 0,992 | 19,604 | 38,986 | 1095 | 0,012 | Protein coding |
| 12 | TUBA1B | -1,074 | 637,535 | 302,932 | 17628 | 0,005 | Protein coding |
| 9 | TXN | -1,086 | 84,892 | 39,984 | 3262 | 0,035 | Protein coding |
| 12 | TXNRD1 | -1,251 | 436,283 | 183,346 | 14700 | 0,018 | Protein coding |
| 13 | UBAC2 | 0,759 | 86,855 | 147,019 | 5574 | 0,013 | Protein coding |
| 7 | UBE2H | 0,786 | 423,283 | 729,788 | 26793 | 0,004 | Protein coding |
| 1 | UBE2T | -1,026 | 13,923 | 6,838 | 455 | 0,034 | Protein coding |
| 2 | USP37 | -0,999 | 47,324 | 23,672 | 1514 | 0,044 | Protein coding |
| 3 | VPS8 | 0,844 | 86,422 | 155,156 | 5786 | 0,021 | Protein coding |
| 6 | WDR27 | 1,599 | 4,936 | 14,95 | 464 | 0,043 | Protein coding |
| 18 | ZNF519 | 2,015 | 2,159 | 8,727 | 182 | 0,047 | Protein coding |
| 19 | MIR4750 | 3,182 | 0,038 | 0,341 | 14 | 0,038 | microRNA |
| 2 | AC010980.2 | -2,519 | 10,475 | 1,827 | 302 | 0,012 | lincRNA |
| 13 | DLEU1 | -1,382 | 13,892 | 5,331 | 258 | 0,03 | lincRNA |
| 18 | GATA6-AS1 | 1,167 | 43,036 | 96,632 | 3260 | 0,002 | lincRNA |
| 6 | LINC00473 | -4,486 | 8,439 | 0,377 | 148 | 0,024 | lincRNA |
| 17 | MIR22HG | -1,424 | 314,599 | 117,257 | 9397 | 0,011 | lincRNA |
