# Supplementary material for: A comprehensive method protocol for annotation and integrated functional understanding of lncRNAs
Source: Brief Bioinform. 2019 Oct 3;21(4):1391–6. doi: 10.1093/bib/bbz066 (PMC7373182; doi:10.1093/bib/bbz066)
Supplement: Supplementary_Table_S5_bbz066 [file supplementary_table_s5_bbz066.docx]

| Term ID | Name | p-value |
| --- | --- | --- |
| HP:0001627 | Abnormal heart morphology | 0^1^ |
| HP:0001635 | Congestive heart failure | 0 |
| ORPHANET:228410 | Polyvalvular heart disease syndrome | 0 |
| OMIM:614980 | CONGENITAL HEART DEFECTS, MULTIPLE TYPES, 2 | 0 |
| OMIM:217085 | CONGENITAL HEART DEFECTS, HAMARTOMAS OF TONGUE, AND POLYSYNDACTYLY | 0 |
| ORPHANET:1338 | Heart defect-tongue hamartoma-polysyndactyly syndrome | 0 |
| HP:0030680 | Abnormality of cardiovascular system morphology | 0 |
| HP:0001639 | Hypertrophic cardiomyopathy | 0 |
| HP:0001645 | Sudden cardiac death | 0 |
| ORPHANET:3286 | Catecholaminergic polymorphic ventricular tachycardia | 0 |
| HP:0031319 | Cardiomyocyte hypertrophy | 0 |
| OMIM:617223 | SUDDEN CARDIAC FAILURE, ALCOHOL-INDUCED | 0 |
| OMIM:610181 | AICARDI-GOUTIERES SYNDROME 2 | 0 |
| OMIM:616117 | CARDIAC CONDUCTION DISEASE WITH OR WITHOUT DILATED CARDIOMYOPATHY | 0 |
| OMIM:613172 | CARDIOMYOPATHY, DILATED, 1DD | 0 |
| OMIM:616789 | MENTAL RETARDATION AND DISTINCTIVE FACIAL FEATURES WITH OR WITHOUT CARDIAC DEFECTS | 0 |
| OMIM:614916 | VENTRICULAR TACHYCARDIA, CATECHOLAMINERGIC POLYMORPHIC, 4 | 0 |
| OMIM:617222 | SUDDEN CARDIAC FAILURE, INFANTILE | 0 |
| HP:0011025 | Abnormality of cardiovascular system physiology | 0 |
| ORPHANET:263297 | Glycogen storage disease with severe cardiomyopathy due to glycogenin deficiency | 0 |
| OMIM:615010 | AICARDI-GOUTIERES SYNDROME 6 | 0 |
| OMIM:616500 | CARDIOENCEPHALOMYOPATHY, FATAL INFANTILE, DUE TO CYTOCHROME c OXIDASE DEFICIENCY 3 | 0 |
| OMIM:613255 | CARDIOMYOPATHY, FAMILIAL HYPERTROPHIC, 15 | 0 |
| OMIM:611407 | CARDIOMYOPATHY, DILATED, 1W | 0 |
| OMIM:605362 | CARDIOMYOPATHY, DILATED, 1J | 0 |
| OMIM:600919 | CARDIAC ARRHYTHMIA, ANKYRIN-B-RELATED | 0 |
| ORPHANET:217622 | Sensorineural deafness with dilated cardiomyopathy | 0 |
| HP:0001671 | Abnormal cardiac septum morphology | 0 |
| HP:0030872 | Abnormal cardiac ventricular function | 0 |
| HP:0004756 | Ventricular tachycardia | 0.001 |
| ORPHANET:51 | Aicardi-GoutiÃ¨res syndrome | 0.001 |
| HP:0001706 | Endocardial fibroelastosis | 0.001 |
| HP:0001688 | Sinus bradycardia | 0.001 |
| HP:0001640 | Cardiomegaly | 0.001 |
| HP:0001649 | Tachycardia | 0.001 |
| HP:0001662 | Bradycardia | 0.002 |
| HP:0011664 | Left ventricular noncompaction cardiomyopathy | 0.003 |
| HP:0011852 | Chylopericardium | 0.003 |
| HP:0001626 | Abnormality of the cardiovascular system | 0.003 |
| HP:0001644 | Dilated cardiomyopathy | 0.003 |
| HP:0001651 | Dextrocardia | 0.013 |
| HP:0001695 | Cardiac arrest | 0.013 |
| HP:0012819 | Myocarditis | 0.02 |
| HP:0001638 | Cardiomyopathy | 0.026 |
| hsa04261 | Adrenergic signaling in cardiomyocytes | 0 |
| WP4300 | Extracellular vesicles in the crosstalk of cardiac cells | 0 |
| WP1544 | MicroRNAs in cardiomyocyte hypertrophy | 0 |
| WP536 | Calcium Regulation in the Cardiac Cell | 0 |
| WP1559 | TFs Regulate miRNAs related to cardiac hypertrophy | 0 |
| WP2795 | Cardiac Hypertrophic Response | 0 |
| WP3668 | Hypothesized Pathways in Pathogenesis of Cardiovascular Disease | 0 |
| hsa04260 | Cardiac muscle contraction | 0.003 |
| hsa05416 | Viral myocarditis | 0.037 |
| WP1528 | Physiological and Pathological Hypertrophy of the Heart | 0 |
| WP1591 | Heart Development | 0 |

^1^”0” is mathematically speaking not reached, but indicated a p values < 10^-30^
