# Supplementary material for: A comprehensive method protocol for annotation and integrated functional understanding of lncRNAs
Source: Brief Bioinform. 2019 Oct 3;21(4):1391–6. doi: 10.1093/bib/bbz066 (PMC7373182; doi:10.1093/bib/bbz066)
Supplement: Supplementary_Table_S6_2nd_revision_bbz066 [file supplementary_table_s6_2nd_revision_bbz066.docx]

**Supplementary Table S6a**

| Name of mRNA | expression (logFC)^1^ |
| --- | --- |
| ATXN2 | - |
| BRF1 | - |
| DDHD2 | - |
| LETM1 | - |
| LTBP3 | 0.955 |
| MAP3K1 | - |
| MFHAS1 | - |
| MGAT4B | - |
| NDFIP1 | - |
| NLGN2 | - |

Interaction prediction of LTBP3 and GATA6-AS using IntaRNA

ENST00000301873.9

137 171

| |

5'-CUU...UCCC G C G UACC...UUA-3'

GCCC CUUCCCC CGCUGCC UCC CUCGCU CCCCC

|||| ||:|||| |:||||| :|| |:|||| |||||

CGGG GAGGGGG GUGACGG GGG GGGCGA GGGGG

3'-GAC...GGAA G C UCC ACA UCCU...CAA-5'

| |

706 667

18_GATA6-AS1

interaction energy = -37.6948 kcal/mol

**Supplementary Table S6b**

| Name of mRNA | expression (logFC) | shared miRNA interaction |
| --- | --- | --- |
| ALG5 | -0.896 | MIR6088 |
| ANKHD1-EIF4EBP3 | 2.509 | MIR6088 |
| ANKRD29 | 1.124 | MIR181D |
| ATP1A1 | -1.094 | MIR6835 |
| CARS | -0.864 | MIR149, MIR6088 |
| CCNG1 | -1.023 | MIR181D |
| CCNG2 | 1.035 | MIR6835 |
| CDKN3 | -2.116 | MIR181D |
| COL4A5 | 0.82 | MIR149 |
| DMKN | 2.031 | MIR149, MIR6835 |
| DTL | -1.665 | MIR149 |
| EIF4G1 | -0.946 | MIR615 |
| EMP3 | -0.95 | MIR6835 |
| FKBP11 | -1.088 | MIR6835 |
| FOXK2 | -0.884 | MIR6835 |
| H2AFZ | -1.141 | MIR6835 |
| HJURP | -4.987 | MIR149 |
| IGF2BP2 | -1.015 | MIR615 |
| LPIN1 | -0.81 | MIR6835 |
| LTBP3 | 0.955 | MIR149, MIR615 |
| MYO9A | -0.916 | MIR149 |
| PAN2 | 0.798 | MIR6835 |
| PCNT | -0.939 | MIR149 |
| PNPLA8 | -1.058 | MIR181D |
| PPDPF | 1.234 | MIR615 |
| RABGAP1L | 0.916 | MIR149, MIR6088 |
| RNASET2 | 1.274 | MIR615 |
| RSPRY1 | -0.865 | MIR6835 |
| SORBS2 | 0.866 | MIR6835, MIR181D |
| SRGAP3 | 1.189 | MIR6088, MIR6835 |
| SRGN | -1.171 | MIR6835, MIR181D |
| STAT3 | -1.847 | MIR181D |
| TFRC | -1.042 | MIR149 |
| TMC6 | 0.889 | MIR149 |
| TPRKB | -0.895 | MIR6835 |
| TXNRD1 | -1.251 | MIR6835 |
| UBE2H | 0.786 | MIR181D |
| UBE2T | -1.026 | MIR6835 |
| USP37 | -0.999 | MIR149 |
| ZNF519 | 2.015 | MIR181D |
