# Supplementary material for: A comprehensive method protocol for annotation and integrated functional understanding of lncRNAs
Source: Brief Bioinform. 2019 Oct 3;21(4):1391–6. doi: 10.1093/bib/bbz066 (PMC7373182; doi:10.1093/bib/bbz066)
Supplement: Supplementary_Table_S7_bbz066 [file supplementary_table_s7_bbz066.docx]

| NAME |
| --- |
| Pax-5 [T00070] |
| ER-alpha [T00261] |
| YY1 [T00915] |
| C/EBPbeta [T00581] |
| FOXP3 [T04280] |
| STAT4 [T01577] |
| AP-2alphaA [T00035] |
| GR-beta [T01920] |
| GR-alpha [T00337] |
| TFIID [T00820] |
| HNF-1A [T00368] |
| HNF-3alpha [T02512] |
| TFII-I [T00824] |
| E2F-1 [T01542] |
| IRF-2 [T01491] |
| c-Ets-1 [T00112] |
| GCF [T00320] |
| GR [T05076] |
| GATA-1 [T00306] |
| XBP-1 [T00902] |
